# The effect of AIDS defining conditions on immunological recovery among patients initiating antiretroviral therapy at Joint Clinical Research Centre, Uganda

**DOI:** 10.1186/1742-6405-6-17

**Published:** 2009-07-24

**Authors:** Brian K Kigozi, Samwel Sumba, Peter Mudyope, Betty Namuddu, Joan Kalyango, Charles Karamagi, Mathew Odere, Elly Katabira, Peter Mugyenyi, Francis Ssali

**Affiliations:** 1 Clinical Epidemiology Unit, Faculty of Medicine, Makerere University College of Health Sciences, P.O. BOX 7072, Kampala, Uganda; 2 Joint Clinical Research Centre, P.O. BOX 10005, Kampala, Uganda; 3 Faculty of Medicine, Makerere University College of Health Sciences, P.O. BOX 7072, Kampala, Uganda

## Abstract

**Background:**

Many HIV-infected patients only access health care once they have developed advanced symptomatic disease resulting from AIDS Defining Conditions (ADCs). We carried out a study to establish the effect of ADCs on immunological recovery among patients initiated on antiretroviral therapy (ART).

**Methods:**

A retrospective cohort of 427 HIV-1 patients who were initiated on ART between January 2002 and December 2006 was studied. Data on ADCs was retrieved from Joint Clinical Research Centre (JCRC) data base and backed up by chart reviews. We employed Kaplan-Meier survival curves to estimate median time to 50 CD4 cells/μl from the baseline value to indicate a good immunological recovery process. Cox proportional hazard models were used at multivariate analysis.

**Results:**

The median time to gaining 50 CD4 cells/μl from the baseline value after ART initiation was longer in the ADC (9.3 months) compared to the non-ADC group (6.9 months) (log rank test, p = 0.027). At multivariate analysis after adjusting for age, sex, baseline CD4 count, baseline HIV viral load, total lymphocyte count and adherence level, factors that shortened the median time to immunological recovery after ART initiation were belonging to the non-ADC group (HR = 1.31; 95% CI: 1.03–1.28, p = 0.028), adherence to ART of ≥ 95% (HR = 2.22; 95% CI: 1.57–3.15, p = 0.001) and a total lymphocyte count ≥ 1200 cells/mm^3 ^(HR = 1.84; 95% CI: 1.22–2.78, p = 0.003). A low baseline CD4 count of ≤ 200 cells/μl (HR = 0.52; 95% CI: 0.37–0.77, p = 0.001) was associated with a longer time to immunological recovery. There was no interaction between low CD4 counts and ADC group.

**Conclusion:**

Patients with ADCs take longer to regain their CD4 counts due to the defect in the immune system. This may prolong their risk of morbidity and mortality.

## Introduction

During 2005, the World Health Organization (WHO) had estimated that there were over 1.3 million people receiving anti retroviral therapy (ART) in low and middle-income countries, representing 20% of 6.5 million estimated to need it [1]. In Uganda, the estimated number of patients on ART is over 135,000 out of 300,000 patients in dire need of treatment and for the past 6 years its use has markedly expanded as part of the ART scale-up that started in 2003 with 30,000 patients[2,3].

As the number of individuals able to access treatment is increasing, one of the challenges facing ART services in sub-Saharan Africa is that many HIV-infected patients only access healthcare once they have developed advanced symptomatic disease resulting from AIDS Defining Conditions (ADCs)[4]. The median CD4 cell count among those enrolling in ART programmes is often very low, which increases morbidity and mortality [4,5]. Advanced pre-treatment immunodeficiency has also been found to be associated with diminished capacity for restoration of CD4 cell counts and CD4 cell functional responses after ART initiation. Therefore, this raises the concern that many ADCs patients entering ART programmes in sub-Saharan Africa may have limited potential for immunological recovery[5,6]. This problem is further compounded by the fact that the number of studies on people with ADCs and immunological recovery are being limited. In this paper we examine the effect of ADCs on immunologic recovery among naive patients initiated on ART at Joint Clinical Research Centre (JCRC), Kampala, Uganda.

## Methods

### Study design and patient selection

This study was a retrospective cohort. JCRC is one of the established centers providing ART and is located in Kampala, the capital city of Uganda. It was founded in 1991 and has been providing anti-retroviral therapy since1996. Combination ART (cART) became available in 1998 but was quite expensive at that time. Generic Fixed Drug cART which was much cheaper(about $50/month) and more accessible by 2002 under the UNAIDS/Ministry of Health (Uganda) HIV Drug Access Initiative Programme [7]. We retrieved data from JCRC data base of all patients who had been initiated on ART between 2002 and 2006. This was backed up by chart review for all charts queried from the data base. During the period the study was done, the data beyond December 2006 was not yet available in the JCRC data set. The patients were ≥ 18 years and ART naive. The exposure of interest was the presence of ADCs at ART initiation among these patients. We classified ADCs according to the WHO clinical staging system [8]. A patient was classified as belonging to the ADC group if was diagnosed with an ADC in the period 12 weeks before or 12 weeks after ART initiation. This classification excludes patients with tuberculosis infections who were on intensive phase of anti-tuberculosis treatment due to the drug interactions and poor virological response [9]. We also excluded patients seen at baseline that had no subsequent follow-up and patients who had previous exposure to dual ART due to poor virological response [10]. The study was approved by the Makerere University College of Health Sciences Research and Ethics Committee. The primary end point was time from ART initiation to immunological recovery, which was defined as the attainment of ≥ 50 CD4 counts/μl above the baseline CD4 cell count that indicated a good immunological recovery process [11,12].

### Diagnostic methods

Diagnosis was done and documented in the charts by the attending physician. This depended on history from the patient, clinical examination, and backed up by laboratory results. HIV wasting syndrome was defined as more than 10% documented loss of body weight and either unexplained chronic diarrhea (> one month), or chronic weakness and unexplained prolonged fever (> one month) with pyrexia recorded on at least on one occasion [8]. Cryptosporidiosis was diagnosed by modified Ziehl Nielsen stain on faecal smear; cryptococcal infection was diagnosed on Indian ink stain of cerebral spinal fluid; muco-cutaneous herpes simplex infection and oesophageal candidiasis were diagnosed clinically; tuberculosis infections were diagnosed by presence of acid fast bacilli from lymph node biopsy, or a pleural effusion with a lymphocytosis which responded to anti-tuberculosis treatment. Kaposi sarcoma was diagnosed histologically, although some cases were diagnosed clinically. Cerebral Toxoplasmosis depended on a positive computer tomography scan, and or a focal neurological disease with a high titre of Ig G antibodies.

### Laboratory assessment

Laboratory measurements included a complete blood cell count, CD4 lymphocyte count, and quantitative measurement of HIV load. Quality control assurance was done in reference to international accredited laboratories; UK National External Quality Assessment Service (UKNEQAS) and College of American Pathologists (CAP).

Viral load count was done with use of Amplicor monitor standard assay, version 1.5 (Roche Molecular Systems), with a minimum detection limit of 400 copies/ml. In 2006, an assay with a detection limit of 50 copies/ml was acquired by the unit. Viral load count was not consistently done.

CD4 lymphocytes were analyzed by flow cytometry (Bendict Dickson, USA). This was periodically done every 6 months during the routine visit to the out-patient clinic. Frequently, this schedule was not routinely followed and depended upon the discretion of the attending physician during the interim visits when patients were sick. The time to immunological recovery was estimated to the nearest clinic visit and CD4 count. We right censored patients if they were lost to follow-up after a period exceeding 12 weeks or when failed to gain 50 CD4 cells/μl above the baseline value. A patient was considered lost to follow-up if did not make contact with JCRC out-patient clinic for a period exceeding 90 days during the study period. Adherence to ART was determined according to the patient's previous four days recall when visited the JCRC out-patient clinic and was considered when the patient took ≥ 95% of the prescribed medications [13].

### Statistical analysis

We aimed to achieve a sample size with a power of 90% to detect the differences between ADC and non-ADC group, and was estimated using formula for survival analysis [14]. We set the level of statistical significance, α at 0.05. The data was queried in MS ACCESS, exported to MS EXCEL and analyzed using STATA (version 8). Kaplan-Meier survival curves were used to estimate median time to immunological recovery. Cox regression hazard models were used to determine factors influencing time to immunological recovery. We fitted a Cox regression models that included all variables which were statistically significant at bivariate analysis. Sex was included in the models because of the previous findings from a study [15]. The interaction between baseline CD4 count and other variables (group status, age, sex, total lymphocyte count, adherence level and viral load) was assessed by performing a Chunk test. At baseline CD4 count of 50, 100, 150, 200, 250 and 350 cells/μl, we compared the -log likelihood of the model with interaction terms to a model without the interaction terms. Interaction was considered when the difference (multiplied by 2) of the -log likelihood was greater than the critical chi-square value of 12.59 at 6 degrees of freedom.

## Results

**Figure 1:**
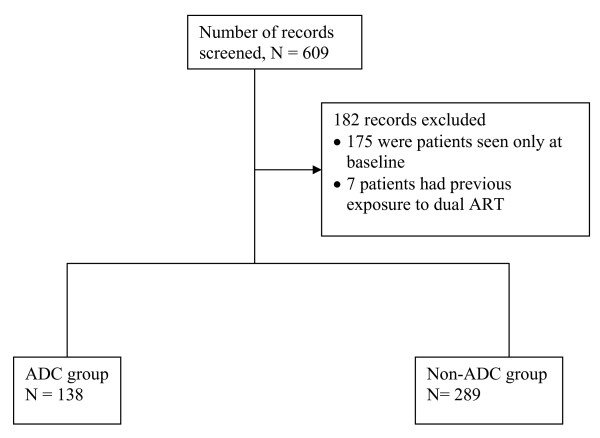
**Study profile of 609 patients initiating ART at JCRC Kampala, during 2002–2006**.

We queried 609 patients from data base who were initiated on antiretroviral therapy between January 1^st ^2002 and December 31^st ^2006, at JCRC out-patient clinic. This number excludes patients who are currently enrolled in ongoing clinical trials and patients who were monitored by physicians outside the JCRC facility but receiving ART at JCRC pharmacy, whose data is stored else where. A total of 182 records were excluded because the patients were seen once at ART initiation and lacked follow-up visits or had previous exposure to dual ART. The remaining 427 patients whose records were used for analysis, had baseline CD4 load count and had been seen on one or more subsequent visits within a period of 12 months follow-up (Figure 1). Out of 413 patients who had a baseline viral load assay, 213 (51.2%) had a second or subsequent viral assay. Sixty nine percent (148/213) achieved a detection limit of 400 or less copies/ml during the first year of ART. Among the patients that achieved viral suppression to a detection limit of 400 or less copies/ml, 68.2% (101/148) belonged to the non-ADC group.

### Descriptive analysis

Of 427 patients who were initiated on ART, 32.3% belonged to the ADC group. The most prevalent ADCs were tuberculosis infections, HIV wasting syndrome, Kaposi sarcoma, *pneumocystis jerovici *pneumonia and cerebral toxoplasmosis (Table 1). The baseline characteristics of 427 patients initiated on ART were comparable in the ADC and non-ADC groups (patients who did not have ADCs at ART initiation), with exception of CD4 counts, adherence level, WHO stage and weight (Table 2). During the follow-up, the mean number of consultations by the patients (due to any infectious cause) at the out-patient clinic in the ADC group was higher than that of the non-ADC group, 16 (SD = 8.5) vs 7 (SD = 4.8), p = 0.020.

**Table 1:** ADC diagnoses among 138 patients initiated on ART at JCRC during 2002–2006

**ADC Diagnoses** **N = 196**	**Frequency**	**Percentage**
Tuberculosis infections	63	32.1
HIV wasting syndrome	51	26.0
Kaposi sarcoma	21	10.7
*Pnuemocystis jerovici *pneumonia	17	8.7
Toxoplasmosis	15	7.7
Cryptococcal meningitis	12	6.1
Esophageal candidiasis	10	5.1
* Others	7	3.6

* 2 CMV retinitis, 2 HSV mucocutaneous infection > 1 month, 1 lymphoma, 1 cryptosporidial diarrhea, 1 MAC infection

**Table 2:** Baseline comparison of socio-demographic and clinical characteristics of 427 patients initiated on ART at JCRC, during 2002–2006

**Variables**	**ADC group** **N = 138**	**Non-ADC group** **N = 289**	**p-value**
**Sex (%)**			
Male	59(42.8)	103(35.3)	0.137
Female	79(57.2)	187(64.7)	
**Age (years)**			
Mean (SD)	40(11)	40(10)	0.331
Occupation (%)*			
Salaried	36(27.1)	105(37.5)	0.185
Non-salaried	55(41.4)	106(37.9)	
Peasant	6(4.5)	11(3.9)	
Unemployed	36(27.1)	58(20.7)	
Education (%)*			
None	24(17.8)	54(19.1)	0.380
Primary	51(37.8)	88(30.9)	
Post-Primary	60(44.4)	142(50.0)	
Residence by district (%)*			
Inside Kampala	78(58.7)	136(51.5)	0.178
Outside Kampala	55(41.4)	128(48.5)	
Marital status (%)*			
Married	57(42.5)	105(38.8)	0.818
Single	40(29.9)	88(32.5)	
Widowed	23(17.2)	53(19.6)	
Separated/divorced	14(10.5)	25(9.1)	
**CD4 (cells/μl)**			
Median (IQR)	74(28, 75)	147(64, 240)	**0.001**
**Viral load (copies log10/ml)**			
Median (IQR)	6.8(6.0, 12.1)	6.0(6.0, 11.6)	0.256
**TLC^† ^(cells/mm^3^) × 10^3^**			
Median (IQR)	1.8(1.3, 2.6)	1.9(1.3, 2.6)	0.409
**Hemoglobin (g/dl)**			
Median (IQR)	11.6(10.4, 13.5)	12.1(10.4, 13.5)	0.277
Adherence (%)*			
Median (IQR)	60(0, 100)^a^	95(20, 100)^b^	**0.001**
			
**WHO stage (%)**			
1	0(0)	15(6.6)	**0.001**
2	0(0)	105(46.3)	
3	0(0)	107(47.1)	
4	138(100.0)	0(0)	
**Weight (kg)**			
Median (IQR)	57.0(18.8, 65.8)	60.0(53.0, 72.0)	**0.001**
**ART regimen (%)**			
Non-protease inhibitor-based	119(86.2)	264(91.4)	0.104
Protease inhibitor-based	19(13.8)	25(8.6)	

* **Missing values**

**TLC**^† ^= Total Lymphocyte Count

^a ^Proportion of patients adherent to ART in the ADC group;12.3%(17/138)

^b ^Proportion of patients adherent to ART in the non-ADC group;65.6%(187/285)

### Bivariate analysis of socio-demographic and clinical factors

**Figure 2:**
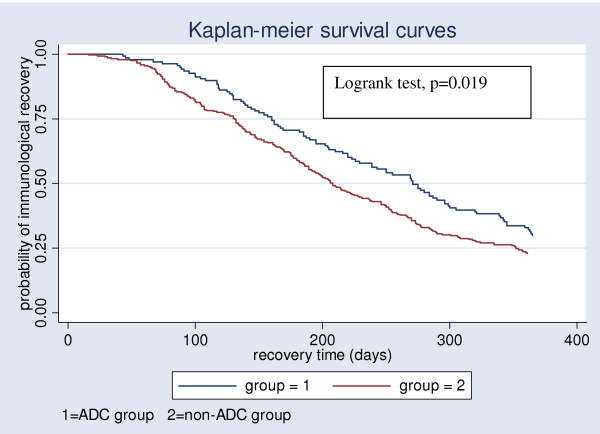
**Kaplan-Meier survival curves comparing time to immunological recovery among 427 patients during ART initiation at JCRC during 2002–2006**.

We studied 427 patients who contributed a total of 123,094 patient-days of follow-up. Three hundred and twenty (74.9%) patients attained immunological recovery during the study period, giving an overall immunological recovery rate of 2.5 per 1000 patient-days. The proportions of patients achieving immunological recovery were 13.0% (18/138), 39.9% (55/138), 56.5% (78/138), 71.7% (99/138) in the ADC group at 3, 6, 9 and 12 months respectively. For the non-ADC group, the proportion of patients achieving immunological recovery were 13.1% (38/289), 40.8% (118/289), 64.0% (185/289), 77.2% (223/289) at 3, 6, 9 and 12 months respectively. The median time to immunological recovery in the ADC group was longer than that of the non-ADC group (271 vs 208 days), (logrank test, p = 0.019) (Figure 2).

Old age (> 40 years), having no ADC at ART initiation, a baseline total lymphocyte count of ≥ 1200 cells/mm^3 ^and adherence to ART (≥ 95%) were associated with reduced time to attain immunological recovery. A low baseline CD4 count (≤ 200 cells/μl) and a high viral load count (≥ 6.0 log copies/ml) were associated with a longer time to attain immunological recovery (Table 3).

**Table 3:** Bivariate analysis of socio-demographic and clinical characteristics that affect time to immunological recovery among 427 patients initiated on ART at JCRC, during 2002–2006

**Variables**	**Unadjusted** **hazard ratio**	**95% CI**	**p-value**
**Sex**			
Male	1.00		
Female	1.02	0.81–1.28	0.879
**Age (years)**			
≤ 40	1.00		
> 40	1.25	1.01–1.56	**0.046**
			
**Group status**			
ADC	1.00		
Non-ADC	1.33	1.05–1.69	**0.020**
**CD4 (cells/μl)**			
>200	1.00		
≤ 200	0.77	0.60–0.98	**0.034**
			
**Viral load (log 10 copies/ml)**			
< 6.0	1.00		
≥ 6.0	0.93	0.88–0.98	**0.007**
			
**Hemoglobin (g/dl)**			
≤ 10.0	1.00		
> 10.0	0.87	0.64–1.19	0.387
			
**Prophylaxis**	1.00		
Cotrimoxazole	0.97	0.51–1.83	0.926
Dapsone	0.82	0.58–1.16	0.274
Fluconazole	0.97	0.72–1.30	0.830
None			
**Adherence level (%)**			
<95	1.00		
≥ 95	2.31	1.84–2.90	**0.001**
**Weight (kg)**			
≤ 60.0	1.00		
> 60.0	0.81	0.64–1.01	0.067
**ART regimen**			
Non-protease inhibitor-based	1.00		
Protease inhibitor-based	0.86	0.59–1.26	0.446
**Total lymphocyte count (cells/mm^3^) × 10^3^**			
< 1.2	1.00		
≥ 1.2	1.55	1.04–2.30	**0.031**

### Multivariate analysis

The time to attain immunological recovery decreased among patients with no ADCs, a total lymphocyte count of ≥ 1200 cells/mm^3 ^and those with adherence to ART was equal or greater than 95%. A low baseline CD4 count of ≤ 200 cells/μl at ART initiation was associated with a longer time to immunological recovery (Table 4). There was no interaction between the baseline CD4 counts and other variables (group status, age, sex, baseline viral load, baseline total lymphocytes) at 50, 100, 150, 200, 250 and 350 cells/μl.

**Table 4:** Multivariate analysis of factors that affect time to immunological recovery among 427 patients initiating ART at JCRC, during 2002–2006

**Variables**	**Adjusted** **Hazard ratio**	**95% CI**	**p-value**
**Group status**			
Non-ADC	1.00		
ADC	1.31	1.03–1.28	**0.028**
			
**CD4(cells/μl)**			
>200	1.00		
≤ 200	0.52	0.37–0.77	**0.001**
			
**Viral load(copies log10/ml)**			
< 6.0	1.00		
≥ 6.0	0.91	0.90–1.04	0.806
			
**Age(years)**			
≤ 40	1.00		
> 40	1.11	0.81–1.67	0.504
			
**Sex**			
Male	1.00		
Female	1.18	0.85–1.67	0.334
			
**Adherence level(%)**			
< 95	1.00		
≥ 95	2.22	1.57–3.15	**0.001**
			
**Total lymphocyte count (cells/mm^3^) × 10^3^**			
<1200	1.00		
≥ 1200	1.84	1.22–2.78	**0.003**

## Discussion

In our study we found that the median time to immunological recovery after ART initiation was 271 days (9.3 months) in the ADC group compared to 208 days (6.9 months) in the non-ADC group. The ADC group status was associated with a longer median time to immunological recovery after ART initiation in comparison to the non-ADC group. Factors that were independently associated with time to immunological recovery were group status (presence or absence of ADC), baseline CD4 count, baseline total lymphocyte count and adherence to ART. Although, immunological responses to ART have been estimated to be at least an increase of 50–100 CD4 cells/μl/year after ART initiation, the median time to attain this CD4 cell threshold has not been documented [12,16]. We took the worst case scenario and estimated a gain of at least 50 CD4 cells/μl/year from the baseline value to indicate good immunological recovery process.

The differences in median times to immunological recovery between the ADC and non-ADC group found in our study may be a result of profound pre-treatment immuno-suppression which is a common feature of ADCs. The ADCs cause a loss of CD4 memory cells that represent a component of the T-cell repertoire that is specific to most ADC infections. The defect in the CD4 memory cells as a result of ADCs accounts for the inability of patients with ADCs to respond to recall antigens and evoke immunological responses leading to a slower immunological recovery process compared to patients without ADCs [17]. Another explanation could be that the presence of ADCs causes immune activation that leads to increased infection of CD4 cells by HIV virus. This leads into CD4 cell destruction and increased viral replication [18]. Our findings seem to support these findings and are similar to other studies in a multi-centre clinical trial where patients with ADCs who were followed-up for 144 weeks who had a slower immunological recovery than patients without ADCs [19]. The contrasting feature of this study from our study is that it did not determine the median time to immunological recovery. We found that the hazard of attaining immunological recovery when a patient had no ADC increased by 1.3 times compared to when a patient had an ADC.

Other determinants that favored a good immunological recovery process after ART initiation were a baseline CD4 count (> 200 cells/μl), baseline total lymphocytes count (≥ 1200 cells/mm^3^) and adherence to ART (≥ 95%). The hazard of attaining immunological recovery reduced when comparing a patient with ≤ 200 CD4 cells/μl to a patient with > 200 cells/μl by 50%. Our results are consistent with other studies where immunological recovery is largely dependent on baseline CD4 count and thus the timing of ART initiation is important in order to maximize the CD4+ T-cell response to therapy [19]. In contrast, one study from South African community cohort that followed-up patients for 44 weeks, found that patients who had a baseline CD4 count below or equal to 50 cells/μl had equivalent or greater immunological recovery compared to patients with higher baseline CD4 count [16]. We found out that there was no interaction between CD4 count and other variables (group status, viral load count, total lymphocyte count, age, sex), although it has been documented else where [20,21]. The lack of interaction may be a result of inadequate power due to limited sample size among the various CD4 count strata.

We found a significant association between adherence level and time to immunological recovery. The hazard of attaining immunological recovery when a patient had adherence level of ≥ 95% compared to one of lower adherence level was 2.2. The adherence level to ART in the ADC group was much lower than the non-ADC group. Similarly, the proportion of patients who achieved virological suppression in the ADC group was lower than the non-ADC group. The non-adherence in the ADC group could have resulted from the pill burden as a result of the concomitant medications other than the drug-drug interactions from the rifampicin-based and ART regimens which has been reported else where [9]. During the intensive phase of the anti-tuberculosis treatment, patients were started on efavirence instead of niverapine-based regimens, although in some patients ART was deferred until the intensive phase was completed. Another reason for the non-adherence was that the patients were too ill to adhere to ART medications due to the concurrent co-morbidities which is a common feature in the sub-Saharan Africa [22]. Our adherence levels to attain immunological recovery are similar to what other studies have found. In a multi-centre study in Africa ≥ 95% to ART was necessary to achieve viral suppression and increase in CD4 counts [23]. In another study in the USA, adherence of ≥ 95% to protease inhibitors was necessary to achieve a good immunological recovery [24]. During ART initiation, the majority of our patients (89.7%) had been started on non-protease ART regimens.

A baseline total lymphocyte count ≥ 1200 cells/mm^3 ^was found to be associated with a short median time to immunological recovery. The hazard of attaining immunological recovery increased by 1.8 times when comparing a patient with a total lymphocyte count of ≥ 1200 cells/mm^3 ^to one of a lower total lymphocyte count. Some studies have confirmed the significant association between a total lymphocyte count of < 1200 cells/mm^3 ^and subsequent poor immunological recovery, disease progression or mortality [25,26].

In our study, old age was not associated with median time to immunological recovery, although a significant association at bivariate analysis was observed. Later, this association between old age and time to immunological recovery disappeared after adjusting for other variables. Our findings are similar to those in a cohort of older participants in the USA (≥ 55 years) where increasing age was associated with a slower and lower immunological recovery due to age-related decreases in thymopoiesis [27]. The 2 cohorts differed in their mean age. The mean age of the patients in our cohort was 40 years whereas for the cohort in the USA was 55 years.

We also found no effect of sex on median time to immunological recovery. Our findings are similar to other findings else where that show that there are no gender differences between males and females regarding response to ART [28]. On the contrary, one study of antiretroviral-naive patients found that women had a greater CD4 cell response than men, even after adjusting for baseline CD4 cell count and viral load. Pharmacokinetic differences may have resulted in women having higher antiretroviral drug levels, which led to more-profound virus suppression and resulted in a greater increase in CD4 cell count [15].

We recognize our study had the following limitations;

Our study design did not allow for the control of all possible confounders as it was retrospective cohort. We relied on measurements taken in the past and where a possible confounder was not measured we could not control for it. For example, we were not able to determine the effect of Body Mass Index (BMI) as height measurements were only available in about 35 patients. Although the baseline body weights were available, subsequent measurements during the follow-up visits were missing and only available in 40 patients. A baseline BMI of < 20.3 kg/m^2 ^for men and < 18.5 kg/m^2 ^for women is predictive of poor immunological recovery, increased morbidity and mortality [29,30]. We were also not able to determine the effect of payment for ART services as this information was not also available, although we found from the chart reviews that some patients were paying for ART drugs.

The time taken to attain 50 CD4 cells/μl after ART initiation was estimated according nearest date of visit to JCRC outpatient clinic when the CD4 count was done. This estimation could have over or underestimated the true estimate. To minimize errors in estimation of time to attain 50 CD4 cells/μl both the principal investigator and data entrant independently calculated the time, and where differences arose an average was taken. Overall inter-observer kappa statistic was 0.68.

Another bias could have resulted from under diagnosis of ADCs because of lack of diagnostic equipments, a common finding in resource limited settings. It is surprising that some of the ADCs were not diagnosed during 2002–2006 when we reviewed records, whereas had been found to be prevalent by other study sites. For example in a nearby main referral hospital, 18% of hospitalized patients presented with non-typhoid salmonella, as an ADC in blood stream [31]. We minimized under diagnosis of ADC by chart review to compare diagnosis of what was in data base whether conforms to what is in the charts. Another explanation could be that our facility was predominantly an out-patient setting.

Some of the immune responses after ART initiation were likely to have been evoked by immune reconstitution syndrome (IRS) and diagnosed as ADCs. This is an inflammatory response against infectious and non-infectious antigens and usually occurs within 12 weeks after ART initiation [32]. However, only less than 3% of patients had ADCs within 12 weeks of ART initiation, thus ruling out the confounding effect of IRS.

We could also not explain a big loss to follow-up of patients who had enrolled for ART as it was inadequately documented. Of 609 who were eligible and enrolled for ART, 182 (29.9%) could not come for second or subsequent review visits. We could explain the loss follow-up could have resulted from deaths due to profound immuno-suppression at ART initiation. Our findings may have overestimated individuals achieving immunological recovery, as those who died from an AIDS-defining disease shortly after starting ART were excluded from the analysis. It is also possible that these patients sought treatment in other treatment centers elsewhere. Since 2003, Uganda has benefited from roll-out program for ART services where more centers have been accredited to offer HIV services and treatment has been either subsided or become free. The loss to follow-up of 29.9% could have resulted into affecting the generalizability of the study findings. This was minimized by taking precautions to prevent under or over diagnose ADCs by chart review, and correct estimation of time to end points from baseline value by taking independent measurements. Secondly, the baseline characteristics of the patients who were lost to follow-up were similar to the patients who had a subsequent follow-up after the baseline visit (CD4 counts 131 vs 115 cells/μl; p = 0.640). Lastly, findings of this study are strengthened by the relatively homogeneous study population receiving treatment at a single health facility using the national treatment protocols. Patients were all ART-naïve and received a standard triple-drug regimen.

## Conclusion

We can draw the following conclusions from the study;

Patients with ADCs take longer to regain their CD4 counts due to the defect in immune system. This may prolong their risk to morbidity and mortality as a result of opportunistic infections. This paper provides a very strong argument for earlier ART initiation in a Ugandan setting. A key challenge is how to identify and initiate ART among patients with less advanced disease. Early Voluntary Counseling and Testing (VCT), the promotion of VCT within community settings so that people know their HIV sero-status and those who are HIV positive are encouraged so seek early health interventions is one option. Another option would be starting ART at above the threshold of 200 CD4 cells/μl. Recently, the Ugandan ART guidelines has recommended initiating ART at 250 and to 350 cells/μl among pregnant women [3]. The third option would be active surveillance of among patients interfacing with the healthcare system. As previously suggested by Lawn SD et al [4], an obvious target population is those with tuberculosis (TB) and HIV-infected pregnant mothers identified in antenatal clinics (ANCs). These target populations represent a key opportunity for identifying many patients with both advanced and less-advanced immunodeficiency. For example, treatment of pregnant mothers at ANCs is likely not only to result in better outcomes but also to prevent vertical transmission of HIV. Thus, provision of facilities for HIV testing and CD4 cell count measurement at antenatal clinics warrants prioritization as access to ART is increased in low-income countries. Furthermore, although costly, provision of CD4 cell count measurements at voluntary counseling and testing facilities would also help identify other asymptomatic individuals with less-advanced disease who are eligible for ART.

## Competing interests

The authors declare that they have no competing interests.

## Authors' contributions

BKK: Study concept and design, data analysis, interpretation of the study findings and wrote-up the manuscript. SS, MP, BN, CK, EK: Assisted in the interpretation of study findings and the critical revision the manuscript. JK: assisted in data analysis, interpretation of study findings and the critical revision of the final manuscript. MO: Queried and extracted data from JCRC database. PM: assisted in critical revision and the final approval of manuscript. FS: Assisted in the study concept and design, interpretation of the study findings and the critical revision of the manuscript.
